# A preliminary study of the child abuse and central sensitization in adolescent patients with chronic non-organic chest pain and an overlooked condition: juvenile fibromyalgia syndrome

**DOI:** 10.1186/s12969-020-00421-0

**Published:** 2020-03-31

**Authors:** Basak Mansiz-Kaplan, F. Figen Ayhan, Mihriban Cagli, Fatih Atik, İbrahim Ece

**Affiliations:** 1 Ankara Training and Research Hospital, Department of Physical Medicine and Rehabilitation, University of Health Sciences, Ankara, Turkey; 2 School of Medicine, Department of Physical Medicine and Rehabilitation, Usak University, Usak, Turkey; 3 Ankara Training and Research Hospital, Department of Pediatric Cardiology, University of Health Sciences, Ankara, Turkey

**Keywords:** Juvenile fibromyalgia, Child abuse, Central sensitization, Chest pain

## Abstract

**Background:**

Only a small percentage of pediatric chest pain is of cardiac origin and the most common detected cause is musculoskeletal. Among musculoskeletal causes, acute chest pain is better described, with the causes of chronic pain not being adequately investigated in the literature. The aim of studuy is to evaluate the musculoskeletal causes of non-cardiac chest pain and investigate the relationship of chest pain with child abuse and central sensitization.

**Methods:**

Patients aged 12 to 18 years presenting with chest pain for at least 3 months were evaluated by a pediatric cardiologist and those without an organic pathology were referred to the physical medicine and rehabilitation clinic. In addition to detailed history and physical examination, juvenile fibromyalgia was questioned according to the 2016 revised diagnostic criteria of the American College of Rheumatology. The visual analog scale (to measure intensity of chest pain), the Central Sensitization Inventory (to evaluate the presence of central sensitization), the Hospital Anxiety Depression Scale (to determine depression and anxiety), the Childhood Trauma Questionnaire (to assess the presence of child abuse) were administered.

**Results:**

The study was completed with 64 patients. Twenty-six percent of patient (*n* = 17) were diagnosed with juvenile fibromyalgia, and central sensitization was detected in 34.4% (*n* = 22). Pain intensity, anxiety, depression and abuse scores were higher in patients with juvenile fibromyalgia than those without juvenile fibromyalgia and in patients with central sensitization compared to those without central sensitization (*p* < 0.001 for both). Higher scores of pain were related with child abuse [beta = 0.763, p < 0.001, (%95 CI, 4.397; 8.841)] and central sensitization of pain [beta = 0.382, *p* = 0.008 (95% CI: (0.986;6.231)] in regression analyses.

**Conclusion:**

In this study, juvenile fibromyalgia was detected as a cause of non-cardiac chest pain. Juvenile fibromyalgia or central sensitization may also indicate childhood abuse.

## Background

Chest pain is a common complaint of referrals to the cardiology clinic in the pediatric population. However, only 1% of patients presenting to pediatric cardiology with chest pain have a true cardiac pathology, with 99% being of non-cardiac origin. The most common detectable cause is musculoskeletal, although pulmonary, gastrointestinal, psychogenic and idiopathic factors can also be seen [1, 2].

The most common musculoskeletal causes of chest pain are costochondritis, muscular strain, rib fracture, and trauma, which all cause acute pain. It is reported in the literature that idiopathic chest pain has a longer symptom duration than costochondritis-induced chest pain [3]. This suggests that the causes of chronic non-cardiac chest pain require further investigation. In a study evaluating the musculoskeletal system in non-cardiac chest pain, Daskapan et al. noted that one cause of non-cardiac chest pain might be related with thoracic hyperkyphosis, and posture should be evaluated [4].

Juvenile chronic musculoskeletal pain is characterized with pain lasting at least 3 months with reported frequencies as much as 5–20%. Of these children, 25–40% meet the criteria of juvenile fibromyalgia (JFM), which is characterized by chronic diffuse pain and accompanying fatigue and sleep, sensory, autonomic and cognitive disorders [5]. JFM has an incidence of 2.1–6% and is mostly seen in adolescent girls [6]. Reduced physical activity, school absenteeism, and poor problem solving skills are frequently observed. Anxiety is one of the more common symptoms of JFM, but mood disorders can also accompany this condition [7]. In the literature, JFM has not been investigated as one of the causes of non-cardiac chest pain. In addition, there are no studies assessing the relationship of chronic non-cardiac chest pain with central sensitization. There was only one study that evaluated association between unexplained chest pain and child abuse. In thus study, Eslick et al. reported that emotional abuse is a risk factor for having chest pain [8]. We aimed to evaluate the presence of JFM in the differential diagnosis of chronic non-cardiac chest pain and to investigate the role of central sensitization and child abuse in adolescent patients in this preliminary study.

## Methods

### Study design and subjects

**Fig. 1:**
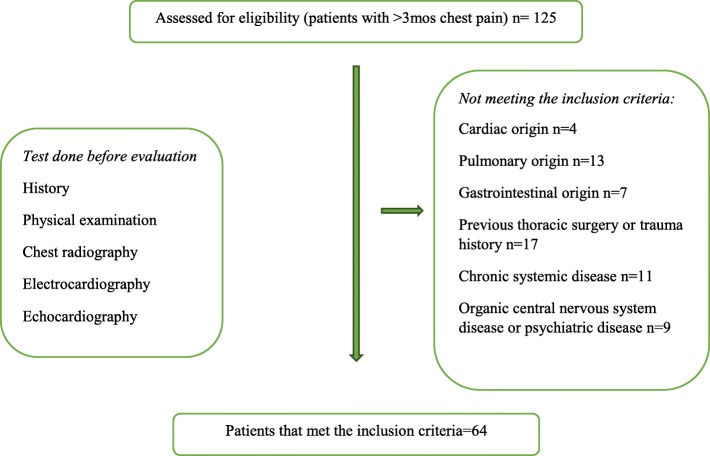
Study flowchart

This prospective study was approved by the ethics committee of the tertiary care hospital. Written consent was obtained from all children who participated in the study and their parents. Patients aged 12 to 18 years presenting to the pediatric cardiology clinic from January 2018 to October 2019 with the complaint of chest pain lasting longer than 3 months without cardiac, pulmonary and gastrointestinal causes were included in the study. All patients underwent a detailed cardiac evaluation, including history, physical examination, chest radiography, electrocardiography, echocardiography and exercise test by the same physician. For those with suspected cardiac origin, the biomarker tests of myocardial injury were also undertaken. The patients with any cardiac or gastrointestinal or pulmonary diseases were excluded from the present study. Those with previous thoracic surgery, trauma history, chronic systemic disease, organic central nervous system disease, mental retardation, and known psychiatric disease or receiving anxiety or antidepressant/psychotropic medications prior to or for the duration of study enrollment were also excluded. Figure 1 presents the flowchart of the study.

After the evaluation of pediatric cardiologist, the detailed history was obtained and a physical examination was performed by the same physical medicine and rehabilitation physician to investigate musculoskeletal diseases. The characteristics of the pain profile such as morning stiffness, nighttime pain, fever, inflammation, and other red flags for pain, and factors associated with increasing and decreasing of pain were questioned. During the physical examination, the chest wall and joints were palpated. In patients detected to have a postural disorder, a radiographic examination of the spine (cervical, thoracic, lumbar and/or scoliosis X-rays) was also evaluated. All patients were evaluated the presence of generalized hypermobility or Ehler-Danlos Syndrome [9]. For those with inflammatory pain, the necessary analyses and examinations (routine blood biochemistry, complete blood count, erythrocyte sedimentation rate per hour, C-reactant protein, sacroiliac X-ray, etc.) were undertaken. The presence of fibromyalgia syndrome was evaluated in patients without these musculoskeletal pathology according to the 2016 revised version of the 2010 American College of Rheumatology (ACR) fibromyalgia diagnostic criteria [10] since this criteria are recommended for the diagnosis of JFM in the adolescent age group [11].

### Outcome measures

After the diagnostic procedures, *the 10-cm Visual Analog Scale (VAS) for pain* was administered to all patients to determine the intensity of chest pain. VAS is a Likert-type scale, with a score ranging from 0 to 10 to evaluate pain intensity. A score of 0 indicates no pain while 10 refers to the most severe pain.

*The Central Sensitization Inventory (CSI)* was used to evaluate the presence of central sensitization. CSI, which can be applied in the presence of chronic pain, is especially utilized in central sensitization syndromes, including fibromyalgia. CSI consists of two parts: Part A is a Likert-type scale that contains 25 questions related to the frequency of health-related symptoms from never (a score of 0) to always (a score of 4). The total score ranges from 0 to 100, and a high score is associated with a high degree of central sensitization. A score of 40 or higher indicates the presence of central sensitization. Part B investigates whether the patients were previously diagnosed with any of the central sensitization syndromes [12]. The validity and reliability studies of the Turkish version of CSI have been previously undertaken [13].

*The Hospital Anxiety and Depression Scale (HADS)* was used to determine the presence of anxiety and depression. HADS consists of a total of 14 items, of which seven question depression and seven anxiety. Each item is scored from 0 to 3. The items related to depression and those related to anxiety are summed separately. In each section, 0–7 is scored as normal, 8–10 as borderline abnormal, and 11 and above as abnormal values [14]. Aydemir et al. conducted the validity and reliability studies of the Turkish version of HADS [15].

*The Childhood Trauma Questionnaire (CTQ)*, developed by Berstein et al., was applied to evaluate childhood abuse. CTQ consists of 40 items based on a Likert scale, scored from 1 (never) to 5 (very often). The minimum and maximum scores are 40 and 200, respectively, with high scores indicating a higher frequency of abuse. CTQ has three subscales evaluating emotional abuse (19 items), physical abuse (16 items), and sexual abuse (five items) [16]. The validity and reliability of the Turkish version of CTQ were investigated by Aslan et al. [17].

All of these questionnaires were administered to the patients individually in a comfortable environment through face-to-face interviews by the same physician.

### Statistical analysis

Statistical analysis was performed using the Statistical Package for the Social Sciences software (SPSS version 23.0, IBM, Armonk, NY, USA). The normal distribution was evaluated using visual and statistical methods. Nominal variables were given as numbers and percentiles, and quantitative data as mean and standard deviation when the data was normally distributed and as median and interquartile ranges if the data distribution was not normal. For the comparison of the two groups, the independent samples t-test was conducted for quantitative data in the presence of normal distribution and the Mann-Whitney U test for the data without normal distribution. To evaluate the correlation between the variables, Pearson’s correlation and Spearman’s correlation analyses were undertaken for the normally and non-normally distributed data, respectively. A regression analysis was performed to predict the correlated data. Significance was evaluated at *p* < 0.05.

## Results

Totally, 125 adolescent patients were analyzed to inclusion criteria and 64 patients were included into the present study. The mean age of included 54 girls and 10 boys was 15.0 ± 1.8 years, and the mean age at onset of chest pain was 13.3 ± 2.2 years. The median duration of symptoms was 12 (interquartile range 4.25–24) months. No patients reported a family history of psychiatric illness or close family history of fibromyalgia/anxiety, depression, and any family history of non-cardiac chest pain. JFM was detected in 17 patients (26%) in according to the 2016 revised version of the 2010 American College of Rheumatology (ACR) fibromyalgia syndrome diagnostic criteria.

Spine x-rays showed abnormal findings in 21 patients (32%), of whom nine had loss of normal cervical lordosis, 11 had right thoracic scoliosis, and one had dorsal hyper-kyphosis.

In the HADS-anxiety subscale, the scores of 40.6% (*n* = 26) of the patients were evaluated as normal, 17.2% (*n* = 11) as borderline abnormal, and 42.2% (*n* = 27) as abnormal. According to the HADS-depression subscale, the scores of 54.7% (*n* = 35) of the patients were normal, 28.1% (*n* = 18) were borderline abnormal, and 17.2% (*n* = 11) were abnormal.

Among the 17 patients with JFM, the HADS-anxiety scores revealed that all of the patients were in abnormal limits, 17.6% (*n* = 3) were borderline, and 82.4% (*n* = 14) were abnormal, and the HADS-depression scores showed that 11.2% (*n* = 2) of the patients were normal, 41.1% (*n* = 7) were borderline, and 47.1% (*n* = 8) were abnormal.

Central sensitization was detected in 34.4% of patients (*n* = 22) according to CSI. The presence of central sensitization among the 17 JFM patients was 70% (*n* = 12).

According to CTQ, the presences of trauma were detected as physical abuse (43.8%, *n* = 28), emotional abuse (39.1%, *n* = 25), and sexual abuse (6.3%, *n* = 4). In the subgroup analysis of 17 patients with JFM, there was 64.7% (*n* = 11) emotional abuse, 64.7% (*n* = 11) physical abuse and 23.5% (*n* = 4) sexual abuse.

There was no significant difference in age, duration of symptoms, and the scores of CSI, VAS, HADS-anxiety, HADS-depression and CTQ when the patients were compared by gender (*p* > 0.05). All of the 17 patients diagnosed with JFM were female.

**Fig. 2:**
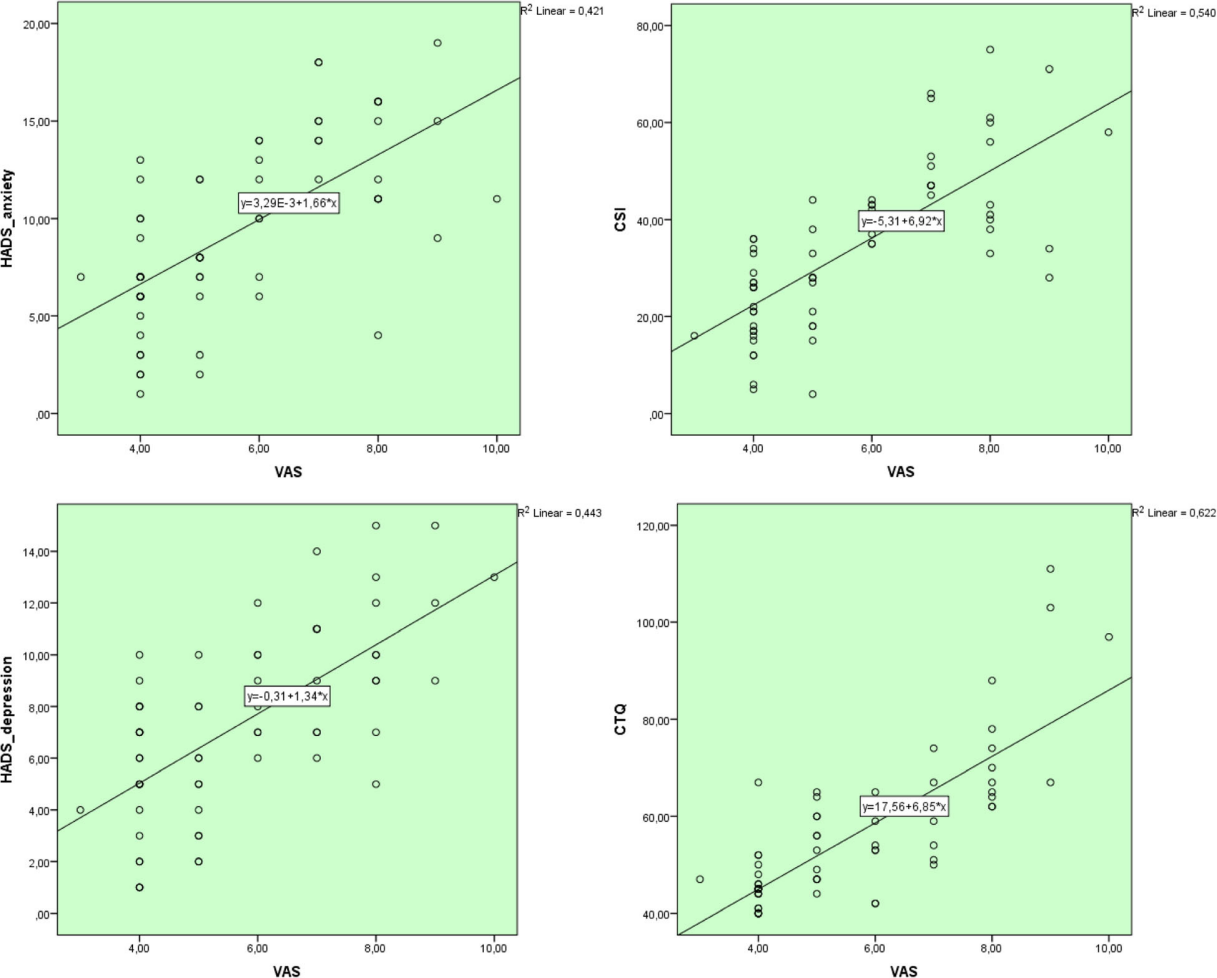
Graphs of the relationship between VAS and HADS-anxiety, HADS-depression, CSI, CTQ

When the patients with and without central sensitization were compared, statistically significant higher VAS, HADS-anxiety, HADS-depression and CTQ scores were found in the central sensitization group (*p* < 0.001) (Table 1). Furthermore, the CSI, VAS, HADS-anxiety, HADS-depression and CTQ scores were significantly higher in patients with JFM than those without JFM (*p* < 0.001) (Table 2). Figure 2 shows the relationship between VAS and HADS-anxiety, HADS-depression, CSI, CTQ.

**Table 1:** Comparison of the patients according to the presence/absence of central sensitization

Central Sensitization	Present (*n* = 22)	Absent (*n* = 42)	P
Age (years)	15.6 ± 1.5	14.6 ± 1.9	0.53^b^
Symptom duration (months)	21 (IQR:6–36)	12 (IQR:4–12.75)	***0.03***^***a***^
VAS	7 (IQR: 6.75–8)	4 (IQR:4–5)	***< 0.001***^***a***^
HADS-anxiety	13.1 ± 3.6	7.4 ± 3.4	***< 0.001***^***b***^
HADS-depression	9.8 ± 2.8	5.9 ± 3.0	***< 0.001***^***b***^
CTQ	66.1 ± 15.9	51.4 ± 11.9	***< 0.001***^***b***^

^a^Mann-WhitneyU test, ^b^independent samples t-test, *VAS* Visual Analog Scale, *HADS* Hospital Anxiety and Depression Scale, *CTQ* Childhood Trauma Questionnaire, *IQR* Interquartile range

**Table 2:** Comparison of the patients according to the presence/absence of juvenile fibromyalgia (JFM)

JFM	Present (*n* = 17)	Absent (*n* = 47)	P
Age (years)	15.2 ± 1.5	14.9 ± 1.9	0.57^b^
Symptom duration (months)	12 (IQR:11–36)	12 (IQR:4–24)	0.67^a^
VAS	8 (IQR:6.5–8)	5 (IQR:4–6)	***< 0.001***^***a***^
HADS-anxiety	13.7 ± 3.1	7.8 ± 3.7	***< 0.001***^***b***^
HADS-depression	10.41 ± 2.3	6.1 ± 3.1	***< 0.001***^***b***^
CSI	49.1 ± 13.4	28.6 ± 13.7	***< 0.001***^***b***^
CTQ	66.4 ± 16.9	52.9 ± 12.7	***0.001***^***b***^

^a^Mann-Whitney U test, ^b^independent samples t-test, *VAS* Visual Analog Scale, *HADS* Hospital Anxiety and Depression Scale, *CTQ* Childhood Trauma Questionnaire, *CSI* Central Sensitization Inventory, *IQR* Interquartile range

A positive correlation was found between CTQ and HADS-anxiety (r = 0.52; *p* < 0.001), HADS-depression (*r* = 0.56; *p* < 0.001), VAS (*r* = 0.77; *p* < 0.001), and CSI (*r* = 0.57; *p* < 0.001). The results of multiple regression analysis for the CTQ are given in Table 3. In predicting CTQ, the model was significant with HADS-anxiety, HADS-depression, CSI and VAS (R^2^ = 0.601, *p* < 0.05), and the only significant predictor was VAS (*p* < 0.001). CSI was positively correlated with HADS-anxiety (*r* = 0.75; *p* < 0.001), HADS-depression (*r* = 0.64; *p* < 0.001), VAS (*r* = 0.75; *p* < 0.001), and CTQ (*r* = 0.57; *p* < 0.001). The results of the regression analysis for the CSI are given in Table 4.

**Table 3:** Results of regression analyses for the Childhood Trauma Questionnaire (CTQ)

**CTQ**	Unstandardized Coefficients		Standardized Coefficients Beta	*P* value	95% CI
	B	SE			
(Constant)	17.305	4.161		0.000	(8.979;25.632)
HADS-Anxiety	0.156	0.423	0.046	0.713	(−0.690;1.003)
HADS-Depression	0.327	0.486	0.076	0.504	(0.646;1.300)
CSI	−0.068	0.131	−0.074	0.606	(−0.330;0.194)
VAS	6.619	1.111	0.763	0.000	(4.397;8.841)

Adjusted R^2^:0.601, *CI* Confidence interval for B, *SE* Standard error, *HADS* Hospital Anxiety and Depression Scale, *VAS* Visual Analog Scale

**Table 4:** Results of regression analyses for the Central Sensitization Inventory (CSI) scores

**CSI**	Unstandardized Coefficients		Standardized Coefficients Beta	*P* value	95% CI
	B	SE			
(Constant)	−3.891	4.659		0.407	(−13.214;5.432)
HADS-Anxiety	1.613	0.363	0.438	0.000	(0.886;2.340)
HADS-Depression	0.815	0.472	0.174	0.090	(−0.130;1.759)
VAS	3.608	1.311	0.383	0.008	(0.986;6.231)
CTQ	−0.067	0.129	−0.061	0.606	(−0.324;0.191)

Adjusted R^2^:0.667, *CI* Confidence interval for B; *SE* Standard error, *HADS* Hospital Anxiety and Depression Scale, *VAS* Visual Analog Scale, *CTQ* Childhood Trauma Questionnaire

## Discussion

This study mainly shows that approximately one of three adolescent with non-cardiac chest pain develop central sensitization associated with fibromyalgia syndrome, and the childhood trauma scores are high in patients with JFM and those with central sensitization. The results of this study also indicate that JFM is one of the causes of pediatric non-cardiac chest pain, and it is necessary to evaluate spinal disorders in these adolescents.

It is well-known that the most common cause of pediatric chest pain is idiopathic, and the most detectable are musculoskeletal causes, including costochondritis, muscle strain, trauma-related pain, and rib fracture. These causes usually result in shorter acute or subacute pain while idiopathic causes lead to chronic pain with a longer duration [3]. In the current study, we evaluated the pediatric population with chest pain lasting for at least 3 months, and the median symptom duration was 12 months. JFM was diagnosed approximately in one in four patients with non-cardiac chest pain (26%, *n* = 17). This suggests that JFM should be investigated, especially in chronic chest pain before evaluating the cause of pain as idiopathic.

Daskapan et al. compared 57 patients with non-cardiac chest pain to a healthy control group. The authors suggested that postural problems could cause non-cardiac chest pain and reported that especially a rounded shoulder, shortness in the pectoralis minor, and thoracic kyphosis played a role in pain [4]. In our study, the pathology detected during postural analysis was confirmed by an X-ray, in addition to physical examination. Of the cases, 32% had a postural spine disorder, indicating the need for a detailed postural and radiographic analysis in patients with non-cardiac chest pain.

It was reported that anxiety is associated with nearly half of the cases presenting with non-cardiac chest pain, while depression is less commonly seen [18]. Khairandish et al. evaluated the presence of anxiety and depression in 194 patients with non-cardiac chest pain in children using the Beck questionnaire, and found significantly higher anxiety (67.5%) and depression (45.9%) in the patient group compared to the control group without chest pain [19]. In another study, 100 adolescent patients with non-cardiac chest pain were compared with the control group and anxiety, depression and suicidal ideation were significantly higher in the patient group [20]. In another study investigating anxiety, depression, and attention deficit and hyperactivity disorder in patients with non-cardiac chest pain, anxiety was found at a rate of 76% and depression at 24% [21]. In our study, similar to the literature, anxiety accompanied with chest pain more frequently than depression. According to the HADS evaluation, normal limits of depression and anxiety were only detected in 54.7 and 40.6% of the patients, respectively.

JFM is a condition characterized by chronic widespread pain. Chest pain is also evaluated as part of the widespread pain index included in the diagnostic criteria of fibromyalgia. The patients with pain lasting longer than 3 months are evaluated in terms of diagnostic criteria [10]. In previous research investigating the causes of non-cardiac chest pain, JFM has not been evaluated as an idiopathic or musculoskeletal factor. In our study, we found JFM at a rate of 26% in adolescent patients presenting with chest pain, and without any cardiac, pulmonary and gastrointestinal pathology.

Central sensitization syndromes including fibromyalgia syndrome are used to describe a group of unexplained disorders without organic pathology [22]. Central sensitization is characterized by an abnormal pain increase in the central nervous system due to neuronal dysregulation and exaggerated excitability, resulting in hypersensitivity to painful and painless stimuli [12]. While there are more studies investigating central sensitization studies in adult fibromyalgia, the studies evaluating presence of central sensitization are very rare in JFM. In a study comparing JFM patients to healthy control, patients with JFM were found more sensitive to pressure pain. They indicated that the results suggest that patients with JFM have a sensitization of peripheral and/or central, like adult fibromyalgia [23]. In the current study, central sensitization values ​​were higher in the group with JFM. This results support recent study and may indicate that central sensitization may be present in JFM patients. The symptom duration was longer, and pain intensity were higher in the group with JFM than controls without JFM. In literature, it was reported that central sensitization may be present with chronic pain especially musculoskeletal pain [24]. In patients who had central sensitization and did not diagnose with JFM, the cause of central sensitization may be chronic chest pain. In addition, in the group with non-cardiac chest pain accompanied by central sensitization, the symptom duration was longer, and pain intensity was higher than controls without central sensitization. This suggests that the presences of JFM and/or central sensitization in these patients may associated with higher pain intensity and longer duration of symptoms.

The HADS-anxiety and HADS-depression scores ​​were higher in the JFM group than in the non-JFM group. Similarly, in the group with central sensitization, these scores ​​were higher compared to those without central sensitization. It was reported that anxiety and depression are commonly seen in JFM. In a study evaluating 91 young adults with the diagnosis of JFM, anxiety was found in 70.3% and depression in 33.3% of patients [25]. In the current study, the anxiety and depression rates were 82.4 and 47.1%, respectively in JFM cases. One reason for our higher rates of anxiety and depression compared to the literature is that rather than diagnostic criteria, we used a screening method (HADS) to determine the presence of these conditions. Another reason is that all of our patients had non-specific chest pain. JFM patients with chest pain may be more likely to have anxiety and depression than those without chest pain. In order to evaluate this situation, further prospective studies comparing these two groups are needed.

Child abuse is a condition that affects mental health and that is often under-reported, under-estimated, and over-looked condition, especially in the adolescent group. If not identified early, it can have chronic consequences [16]. In the literature, it has been reported that as childhood abuse increased, more pain symptoms were observed. And also patients with chronic pain were more likely to report childhood trauma [26]. It has been indicated that there were an association between especially physical and sexual abuse and increasing the risk of chronic pain [27]. In a recent study, women who experienced childhood maltreatment (any of these: physical, sexual, or emotional abuse, neglect), complained with the presence of pain, pain severity, and number of body areas with pain than control group [28]. On the other hand, there is only one study that investigated relationship between abuse and chest pain. Eslick et al. evaluated 27 patients who had an unexplained chest pain and 60 individuals who did not have pain in the last year. They found rates of childhood abuse to be high comparing the control group. They concluded that a history of childhood emotional/verbal is a risk factor for having unexplained chest pain. They demonstrated that emotional abuse was 20.8%, physical abuse was 16.7% and sexual abuse was 34.8% in 27 children with un-explained chest pain [8]. We found higher rates of emotional abuse (39.1%), physical abuse (43.8%) and lower rate of sexual abuse (6.3%) in 64 adolescent patients with non-cardiac chest pain. The possibility of abuse should be considered in the adolescent with non-organic chest pain.

There was limited number of studies investigating relationship between fibromyalgia and childhood abuse in pediatric age group compared to adult fibromyalgia. In a meta-analysis, it has been reported that the association of fibromyalgia with physical and sexual abuse could be confirmed, although level of evidence was poor [29]. In another study evaluating 515 patients with adult fibromyalgia, the adverse childhood experiences were found to associate with fibromyalgia [30]. Hellou et al., assessed the role of childhood abuse in patients with fibromyalgia and compared patients with rheumatic arthritis. They found emotional abuse rates was higher than patients with rheumatic arthritis in fibromyalgia patients [31]. In contrary, Lommel et al. assessed 62 patient in adolescent psychiatric population. Thirty-two of this patients had JFM and they found no connection between JFM and physical or sexual abuse [32]. In our study, the CTQ values were higher in the JFM group compared to the non-JFM group, and there was 64.7% (*n* = 11) emotional abuse, 64.7% (*n* = 11) physical abuse and 23.5% (*n* = 4) sexual abuse in 17 JFM patients. The CTQ values were also higher and in the central sensitization group compared to the patients without central sensitization. In addition, there was a positive correlation between CTQ and the scores of HADS-anxiety, HADS-depression, VAS and CSI. Therefore, we have considered that JFM cases with non-cardiac chest pain should be evaluated for central sensitization and child abuse.

One of the limitations of our study is the small number of patients with JFM. Prospective, controlled studies with a larger number of JFM cases are needed to better evaluate the investigated parameters (central sensitization pain, anxiety, depression, child abuse, etc.) in this age group. Another limitation is that factors such as mental disorders including substance/alcohol dependence, environmental conditions such as family, peers, and school environment and other adverse childhood events (illness, nutrition, economy, or socio-political discrimination) were not explored in this study.

## Conclusion

In adolescent patients presenting with non-cardiac chest pain, JFM in addition to postural/spinal disorders should be evaluated among musculoskeletal causes. JFM associated with central sensitization and child abuse, a previously overlooked cause, should also be investigated in this patient profile. The presence of central sensitization and/or fibromyalgia in children with non-cardiac chest pain may indicate child abuse.

## Data Availability

All data generated or analysed during this study are included in this published article [and its supplementary information files].

## Abbreviations

JFM: Juvenile fibromyalgia
ACR: American College of Rheumatology
VAS: Visual Analog Scale
CSI: Central Sensitization Inventory
HADS: Hospital Anxiety and Depression Scale
CTQ: Childhood Trauma Questionnaire
